# Supplementary material for: Learning curve and surgical outcome of robotic assisted colorectal surgery with ERAS program
Source: Sci Rep. 2022 Nov 29;12:20566. doi: 10.1038/s41598-022-24665-w (PMC9709162; doi:10.1038/s41598-022-24665-w)
Supplement: Supplementary file 2 — Supplementary Information 2. [file 41598_2022_24665_MOESM2_ESM.zip › RAL Raw data-Ñ[▒K/054/HS_ñw╝╨░O▒Kñσ.pdf]

正式摘要(電子簽章：00000209230239)

W52 / W52 ☐英文標題(注意:無英文姓名-請補病人基本檔!) ☐【讀取】憑證

臺中榮民總醫院
出院病歷摘要

轉入號 : 轉出號 :

[redacted]

**(8)**流水編號	
**(9)**入院日期	110/01/18 (2021) 直腸外科 W52 - 092
**(10)**轉科日期	
**(11)**出院日期	110/01/27 (2021) 住院天數計 9 日

診斷

- 入院 :1. Diverticulosis of colon
  2. Cyst over right upper liver (16cm)
- 出院 :1. Diverticulosis of colon, s/p Robot Assisted Colectomy on 2021/01/20
  2. Cyst over right upper liver (16cm), s/p liver Laparoscopic fenestration for hepatic cyst on 2021/01/20

主訴

- Abdominal pain and constipation for months.

病史

- The 80 year old female had history of
  1. Hypertension, under medication
  2. Appendicitis , s/p appendetomy in 1961
  3. Liver cyst
  4. Uterus prolapse, s/p hysterectomy in 2010.
  5. Rupture of right knee ligament, s/p repair surgery

  This time, she suffered from abdominal discomfort and constipation for days. She went to 澄清H for help where abd sono and abd CT showed liver cyst and diverticulosis of colon. Thus, she came to our CRS OPD for help. Conservative treatment was ordered but in vain. After discussion, Robot Assisted Colectomy and liver unroofing of cyst were arranged. This time, she was admitted for scheduled operation.

體檢發現

- 9/28 CT: diverticulum. huge liver cyst. 16 cm.
  - General appearance :
    - easy looking , conscious clear , without cardiopulmonary distress
    - Vital signs自動帶入date 體溫(BT) 脈搏(P) 呼吸(R) 血壓(BP) 身高(BH) 體重(BW) BMI
      20210118 35.3℃ 67次/分 18次/分 139/78mmHg 155.8cm 49.4kg 20.4
    - Vital signs: B.P. 139/78 mmHg , P.R. 67 /min , R.R. 18 /min , B.T. 35.3 ℃
  - HEENT :
    - Eyes: no anemic conjunctiva, no icteric sclera, normal EOM
    - ENT: no otorrhea, no rhinorrhea, no erythematous mucosa, no injected tonsil, no deformity
  - Neck: supple, freely movable, trachea midline,no deformity, no engorged jugular vein , no palpable thyroid or lymph node enlargement
  - Chest:
    - Inspection: Grossly normal, symmetric and fully expansion, no accessory muscle used, no operation scar, no spider angiomata, no gynecomastia
    - Palpation: no palpable mass or nodule, no subcutaneous emphysema, no tenderness
    - Ausculation: clear breathing sound
  - Heart:
    - Percussion: normal heart size

-
  - Ausculation: regular heart beat, normal S1, S2, no gallop, no murmur
- Abdomen:
  - Inspection: in
    - flat contour, symetrically, no scar, no stria, no dilated veins, no rashes
  - Ausculation: normoactive bowel sound, no abdominal bruits
  - palpation: soft, no muscle guarding or rigidity, no local tenderness, no rebounding pain, no palpable mass, no palpable pulsation
  - Percussion: no shifting dulllness, no hepatomegaly, no splenomagaly
-

Peripheral pulsation :	CA	BA	RA	FA	PA	PTA	DPA
Right side :	++	++	++	++	++	++	++/+++
Left side :	++	++	++	++	++	++	++/+++

- Genito-anus: no tenderness or palpable mass lesion or rectal shelf, normal external genitalia, normal anal tone, brown stool

CRS CONDITION

- Imaging studies(Sono , CT of abdomen and chest Films):
  - Diverticulosis of colon, with some small diverticular stones.
-
  - Lung

手術日期及方法

- 手術日期及方法20210120 Liver Laparoscopic fenestration for hepatic cyst
  20210120 1. Robotic (XI) assisted anterior resection.
  # Fenestration for hepatic cyst was done under GS specialist.
  2. Intraoperative colonoscopy : (-)
  3. High ligation (-) : At IMA upper rectum branch. Left colic artery and IMV were preserved.
  4. Take down from the splenic flexure : (-)
  5. Robotic endoGIA (45-4.3) X2 + EEA (29mm) were applied to completed the end to end anastomosis.
  6. Indocyanine green agent was administered for testing during operation.
  7. No drainage tube was inserted.
  8. Hyalobarrier gel was applied below the wound over the LLQ of abdomen.

住院治療經過

- After admission, , pre-op surveyt was done and no abnormal finding. Robot Assisted Colectomy and liver unroofing of cyst was arranged and performed smoothly. She started trying water on 1/22. Foley was removed and she started blant l diet on 2021/1/23. The postoperative course was rather stable and the patient was discharged later under stable condition.

合併症

一般檢查

- 累積報告 :

DATE	TIME	ALB	TP	BIL.T	BIL.D	ALKP	AST	ALT	LDH	CHOL	TG	HDL-C	LDL	UA	NH3	AMY
1100120	2313	3.4		0.58			42	26								
1100125	1022			0.48			18	11								

DATE	TIME	WBC	RBC	HGB	HCT	MCV	PLT	BLST	NEUT	BAND	SEG	LYM	MON	EOS	BAS
1100120	2310	11590		11.5	35.7		166		89.0			6.5	4.4	0.0	0.1
1100125	0937	7110	3.82	12.1	36.2	94.8	172		70.4			19.4	7.5	2.3	0.4

DATE	TIME	NA	K	CL	CA	BUN	CREAT	Mg	P	CRP	ESR	PCT	LIPASE	LACTATE
1100120	2313	142	3.7		7.2	13	0.73							
1100125	1022	143	2.9	107	7.6	8	0.65							

特殊檢查

病理報告

- 活體組織切片病理報告　手術日期：**110/01/20**　病理號：**S1002461**

診斷醫師:文美卿 報告日期:110/01/28
Pathologic diagnosis:

- 1. Cyst, liver, unroofing -- Biliary cystadenoma.
  2. Sigmoid colon, robotic anterior resection --- Diverticulosis.
  3. IMA root, dissection --- Unremarkable fibrovasculoadipose tissue.
  4. Colon, distal cut end, resection --- Congestion.
  Ancillary study for diagnosis:
  Immunohistochemical stains for CK7 and CK19 done in section A. The cystic lining cells are immunoreactive to both CK7 and CK19.
  Gross description:
  The specimen consists of 1) 2 pieces of ruptured cystic tumor, up to 7x2.8x1 cm, labeled as "liver cyst". The wall of cyst is thin and without obvious inner content. 2) a segment of colon measures 11 cm in length, 2.5 cm in circumferential of proximal cut end, and 4 cm in circumferential of distal cut end. There are multiple small diverticulum noted at the mucosa. Other parts of specimen are unremarkable. 3) a piece of tan soft tissue, 1.8x1x0.5 cm, labeled as "IMA root". 4) a piece of tan soft mucosa-covered tissue, 2.3x2x1.3 cm, labeled as "distal cut end". Representative parts for section:
  A-B) liver cyst C-D) bilateral cut ends E-J) lesion; All for section:
  K-L) specimen 3-4.
  #T-59470_2 #M-32700_2 0 1100 000000
  #T-62000_2 #M-81610_2 0 1100 000000

其它

出院時情況

- 3.改門診治療

出院指示

藥名	劑量	劑量單位	用法	頻次	數量	數量單位	現況	天數	單位
Through tab	1	TAB	PO	QN	5	TAB	TH	5	天
Sevikar tab 5/20mg	1	TAB	PO	QD	5	TAB	TH	5	天
Mag. Oxide tab	2	TAB	PO	TID	30	TAB	TH	5	天
Ultracet tab	1	TAB	PO	TID	15	TAB	TH	5	天
Ulstop tab 20mg	1	TAB	PO	BID	10	TAB	TH	5	天

出院計劃

- 門診日期
  - date on 2021/02/01

主治醫師 2222H 林俊余 / 住院醫師 9788J 張廷羽

正式摘要(電子簽章：000000209230229)

W52 / W52

doctorRank:B
